# Prevalence and Antibiotic Resistance of Non-typhoidal *Salmonella* Isolated from Raw Chicken Carcasses of Commercial Broilers and Spent Hens in Tai’an, China

**DOI:** 10.3389/fmicb.2017.02106

**Published:** 2017-10-31

**Authors:** Song Li, Yufa Zhou, Zengmin Miao

**Affiliations:** ^1^College of Basic Medicine, Taishan Medical University, Tai’an, China; ^2^Center for Disease Control, Veterinary Bureau of Daiyue, Tai’an, China; ^3^College of Life Sciences, Taishan Medical University, Tai’an, China

**Keywords:** antibiotic resistance, β-lactamase gene, class 1 integron, *Salmonella*, serotype

## Abstract

The present study was aimed to determine the prevalence and characteristics of *Salmonella* isolated from meat samples of commercial broilers (CB) and spent hens (SH). Between March and June 2016, 200 retail raw chicken carcasses (100 from CB and 100 from SH) were obtained from local supermarkets in Tai’an city of China, and *Salmonella* isolates were then analyzed for antibiotic resistance, serotype, β-lactamase genes, and the presence of class 1 integron. Forty *Salmonella* strains were obtained in this study (CB: 21/100, 21%; SH: 19/100, 19%). Three serotypes were identified in 40 *Salmonella*, and *S.* Enteritidis (CB: 15/21, 71.4%; SH: 10/19, 52.6%) was the dominant serotype, followed by *S.* Typhimurium (CB: 4/21, 19%; SH: 6/19, 31.6%) and *S.* Derby (CB: 2/21, 9.5%; SH: 3/19, 15.8%). Among 21 *Salmonella* isolated from CB, high antibiotic resistance rates were found for ampicillin (20/21, 95.2%), nalidixic acid (18/21, 85.7%), cefotaxime (17/21, 81%), and tetracycline (13/21, 61.9%); class 1 integron was observed in seven isolates (7/21, 33.3%), and gene cassettes included an empty integron (0.15 kb, *n* = 1), *aadA2* (1.2 kb, *n* = 3), *drfA1-aadA1* (1.4 kb, *n* = 1), and *drfA17-aadA5* (1.7 kb, *n* = 2); *bla*_TEM-1_ was the dominant β-lactamase gene (21/21, 100%), followed by *bla*_CTX-M-55_ (7/21, 33.3%). Among 19 *Salmonella* isolated from SH, high antibiotic resistance rates were found for nalidixic acid (19/19, 100%), tetracycline (19/19, 100%), ampicillin (18/19, 94.7%), and ciprofloxacin (13/19, 68.4%); class 1 integron was observed in two isolates (2/19, 10.5%), and gene cassettes included *drfA17-aadA5* (1.7 kb, *n* = 1) and *drfA1-aadA1* (1.4 kb, *n* = 1); *bla*_TEM-1_ was the dominant β-lactamase gene (19/19, 100%), followed by *bla*_CTX-M-55_ (2/19, 10.5%) and *bla*_CMY-2_ (1/19, 5.3%). Collectively, antibiotic-resistant *Salmonella* can be widely detected in retail raw chicken carcasses of CB and SH, and therefore can pose a serious risk to public health.

## Introduction

*Salmonella* is a notorious human pathogen and can lead to acute intestinal disease outbreaks in humans through consumption of contaminated foods (Pegues et al., 2006). It has been widely recognized that poultry products, such as eggs and meats, are a crucial transmission vehicle for *Salmonella* (Kusunoki et al., 2000; Betancor et al., 2010; Painter et al., 2013; Antunes et al., 2016). At present and even for a long time in the future, antibiotic-based treatment for human salmonellosis infection is the most effective method in clinical practice (Ribeiro et al., 2011; Crump et al., 2015). It is therefore pivotal to use antibiotics to prevent and control *Salmonella* infections.

However, the widespread use and even abuse of antibiotics in animal husbandry have facilitated the emergence and dissemination of antibiotic resistance in *Salmonella*, which has posed a serious challenge for the health of animals and humans (Marshall and Levy, 2011; Mukerji et al., 2017). Noticeably, numerous studies in recent years have indicated that extended-spectrum β-lactamase (ESBL)-producing *Salmonella* has been frequently isolated from food-producing animals and animal-derived foods in many countries of the world, including China (Wu et al., 2013; Chon et al., 2015; Franco et al., 2015; Hu et al., 2015; Noda et al., 2015; Ziech et al., 2016; Zhao et al., 2017). ESBL-producing *Salmonella* is able to inactivate and hydrolyze the β-lactam ring in β-lactam antibiotics and third- and fourth-generation cephalosporins, leading to the increase of treatment cost and even to therapy failure, which has triggered a serious public concern (Bonnet, 2004; Pitout and Laupland, 2008). In addition, the class 1 integrons are frequently observed among antibiotic-resistant *Salmonella*, which contributes to the spread of antibiotic resistance genes among bacteria (Wannaprasat et al., 2011).

Therefore, understanding the prevalence and characteristics of *Salmonella* isolated from meat samples of food animal origins is of importance for developing effective treatment strategies to control and prevent *Salmonella* infections in humans and animals. However, information about the occurrence and characteristics of *Salmonella* in chicken meats in China is poorly documented. In China, two main chicken breeds, including introduced commercial broilers (CB) and spent hens (SH), are widely reared and are important sources of chicken meat (Chen et al., 2016). This study was therefore undertaken to determine the prevalence and characteristics of *Salmonella* recovered from retail chicken carcasses of CB and SH in Tai’an region, China.

## Materials and Methods

### Sample Collection

Between March and June 2016, 200 retail raw chicken carcasses without giblets (100 from CB and 100 from SH) were obtained from local supermarkets in Tai’an city, China. These supermarkets had areas of 5,000–10,000 m^2^, offering various foods and household products, in which raw chicken carcasses were sold refrigerated in a meat department. After purchase, the samples were stored in an icebox and immediately transported into our microbiology laboratory for further processing within 24 h.

### *Salmonella* Isolation and Serotype Identification

*Salmonella* isolation was conducted as previously described (Choi et al., 2015). Briefly, each chicken carcass was mixed with 400 ml of buffered peptone water (BPW; Hopebiol, Qingdao, China) contained in a sterile plastic bag to rinse for 1 min by gentle shaking. Twenty-five milliliter of the rinsate was mixed with 25 mL of 2 × BPW and the mixture was incubated overnight at 37°C. A 100 μL aliquot was removed form the BPW enrichment broth and inoculated into Rappaport-Vassiliadis soy peptone broth (10 mL) (RVS; Hopebiol, Qingdao, China), which was incubated for 24 h at 42°C. One loopful of the RVS culture was streaked onto a xylose lysine desoxycholate agar plate (XLD; Hopebiol, Qingdao, China), which was incubated overnight at 37°C. The suspected *Salmonella* colony (red colonies with black centers) on the XLD plates from each chicken meat sample was identified by biochemical confirmation using biochemical tubes (Hopebiol, Qingdao, China), and the results were interpreted according to Bergey’s Manual of Systematic Bacteriology (Garrity et al., 2004).

According to the Kauffmann-White scheme, slide agglutination tests were used to serotype *Salmonella* isolates in this study (S&A Reagents Lab, Bangkok, Thailand).

### Antimicrobial Susceptibility Testing

Based on the guidelines of the Clinical and Laboratory Standards Institute (Clinical and Laboratory Standards Institute [CLSI], 2013), the disk diffusion method was employed to determine antibiotic susceptibilities of *Salmonella* strains. Antibiotics used in this study were amoxicillin/clavulanic acid (20/10 μg), ampicillin (10μg), cefotaxime (30 μg), ciprofloxacin (5 μg), florfenicol (30 μg), gentamicin (10 μg), nalidixic acid (10 μg), spectinomycin (10 μg), tetracycline (30 μg) and sulfamethoxazole/trimethoprim (1.25/23.75 μg) (Hopebiol, Qingdao, China). *Salmonella* strains resistant to no less than three classes of antibiotics were defined as multidrug-resistant (MDR) isolates. *Escherichia coli* ATCC 25922 was used in this study as quality control strain.

### Detection of β-Lactamase Gene

According to the method previously described (Batchelor et al., 2005; Rayamajhi et al., 2008; Li et al., 2013), polymerase chain reaction (PCR) was used to determine the presence of β-lactamase genes (*bla*_TEM_, *bla*_PSE-1_, *bla*_CMY -2_, *bla*_SHV_, *bla*_DHA-1_, *bla*_OXA_, and *bla*_CTX-M_). For isolates carrying *bla*_CTX-M_ genes, *bla*_CTX-M_ gene group was further identified by using PCR (Kim et al., 2015). The PCR products were sequenced (Sunny, Shanghai, China), and the sequences were analyzed and aligned using the NCBI BLAST program^1^.

### Detection of Class I Integrons

Based on the primers previously described, PCR was used to analyze the presence of class 1 integron (Guerra et al., 2001; Kerrn et al., 2002). Additionally, PCR was employed to amplify gene cassettes within the variable region of class 1 integron according to the methods described previously (Sandvang et al., 1998). The amplification fragments were cloned into the pMD18-T vector (Takara, Dalian, China), which were sequenced (Sunny, Shanghai, China).

### Statistical Analyses

Fisher’s exact test was used to compare the prevalence of *Salmonella* and proportions of class 1 integron in *Salmonella* in CB and SH using SPSS 16.0 software (IBM, United States). *P*-values of less than 0.05 were defined as difference significance.

## Results

### *Salmonella* Prevalence

A total of 40 *Salmonella* strains (40/200, 20%) were isolated from the foods, and the prevalence in CB was 21% (21/100) and 19% (19/100) in SH. No significant difference was found in *Salmonella* prevalence between CB and SH samples (*P* > 0.05).

### Serotyping and Antimicrobial Susceptibility Testing

Three serotypes were identified in 40 *Salmonella* strains. *S.* Enteritidis (CB: 15/21, 71.4%; SH: 10/19, 52.6%) was the dominant serotype, followed by *S.* Typhimurium (CB: 4/21, 19%; SH: 6/19, 31.6%) and *S.* Derby (CB: 2/21, 9.5%; SH: 3/19, 15.8%) (**Tables 1**, **2**).

**Table 1:** Antibiotic resistance phenotype, presence of class 1 integron, and β-lactamase genes in *Salmonella* isolated from CB.

Isolates	Serovar	Antibiotic resistance phenotype^a^	Integron/β-lactamase genes
CB-1	*S.* Derby	AMP, CIP, CTX, NAL, TET	*bla*_TEM-1_
CB-12	*S*. Enteritidis	AMP, GEN, CTX, FFC, NAL, TET	(*aadA2*)/*bla*_TEM-1_
CB-14	*S*. Enteritidis	AMP, GEN, CTX, FFC, NAL, TET	*bla*_TEM-1,_ *bla*_CTX-M-55_
CB-18	*S.* Derby	AMP, CTX, NAL	(*drfA1*-*aadA1*)/*bla*_TEM-1_
CB-33	*S*. Enteritidis	AMP, GEN, CTX, FFC, NAL, TET	*bla*_TEM-1,_ *bla*_CTX-M-55_
CB-42	*S.* Typhimurium	AMP, GEN, SPT	(*aadA2*)/*bla*_TEM-1_
CB-48	*S.* Typhimurium	AMP, SPT	*bla*_TEM-1_
CB-49	*S*. Enteritidis	AMP, GEN, CTX, FFC, NAL, TET	*bla*_TEM-1,_ *bla*_CTX-M-55_
CB-51	*S*. Enteritidis	AMP, CTX, NAL	*bla*_TEM-1_
CB-58	*S*. Enteritidis	AMP, GEN, CTX, FFC, NAL, TET	*bla*_TEM-1_
CB-59	*S*. Enteritidis	AMP, CTX, FFC, NAL, TET	*bla*_TEM-1,_ *bla*_CTX-M-55_
CB-63	*S*. Enteritidis	AMP, GEN, CTX, FFC, NAL, TET	(*drfA17-aadA5*)/*bla*_TEM-1,_ *bla*_CTX-M-55_
CB-65	*S*. Enteritidis	AMP, GEN, CTX, FFC, NAL, TET	*bla*_TEM-1_
CB-71	*S*. Enteritidis	AMP, GEN, CTX, FFC, NAL, TET	(*drfA17-aadA5*)/*bla*_TEM-1_
CB-79	*S*. Enteritidis	AMP, GEN, CTX, FFC, NAL, TET	*bla*_TEM-1,_ *bla*_CTX-M-55_
CB-81	*S.* Typhimurium	AMP, SPT	(*aadA2*)/*bla*_TEM-1_
CB-88	*S*. Enteritidis	NAL	*bla*_TEM-1_
CB-89	*S*. Enteritidis	AMP, GEN, CTX, FFC, NAL, TET	*bla*_TEM-1,_ *bla*_CTX-M-55_
CB-91	*S*. Enteritidis	AMP, CIP, CTX, NAL, TET	*bla*_TEM-1_
CB-96	*S*. Enteritidis	AMP, CTX, NAL	empty integron/*bla*_TEM-1_
CB-98	*S.* Typhimurium	AMP, CTX, NAL	*bla*_TEM-1_

^a^*AMC, amoxicillin/clavulanic acid; AMP, ampicillin; CTX, cefotaxime; CIP, ciprofloxacin; FFC, florfenicol; GEN, gentamicin; NAL, nalidixic acid; SPT, spectinomycin; TET, tetracycline; SXT, sulfamethoxazole/trimethoprim*.

**Table 2:** Antibiotic resistance phenotype, presence of class 1 integron, and β-lactamase genes in *Salmonella* isolated from SH.

Isolates	Serovar	Antibiotic resistance phenotype^b^	Integron/β-lactamase genes
SH-3	*S*. Enteritidis	NAL	*bla*_TEM-1_
SH-5	*S.* Enteritidis	AMP, NAL, TET	*bla*_TEM-1_
SH-8	*S.* Enteritidis	AMP, CTX, CIP, NAL, TET	*bla*_TEM-1,_ *bla*_CTX-M-55_
SH-13	*S.* Derby	AMP, CIP, NAL, TET	*bla*_TEM-1_
SH-21	*S.* Enteritidis	AMP, NAL, TET	*bla*_TEM-1_
SH-26	*S.* Enteritidis	AMP, NAL, TET	*bla*_TEM-1_
SH-33	*S.* Enteritidis	AMP, CIP, NAL, TET	*bla*_TEM-1_
SH-35	*S*. Enteritidis	AMP, CIP, CTX, NAL, TET	(*drfA17-aadA5*)/*bla*_TEM-1_
SH-48	*S.* Enteritidis	AMP, CIP, NAL, TET	*bla*_TEM-1_
SH-50	*S.* Enteritidis	AMP, CIP, NAL, TET	*bla*_TEM-1_
SH53	*S.* Derby	AMP, NAL, TET	*bla*_TEM-1_
SH-61	*S.* Typhimurium	AMP, CTX, CIP, NAL, TET	*bla*_TEM-1,_ *bla*_CTX-M-55_
SH-66	*S.* Typhimurium	AMP, CIP, NAL, TET	*bla*_TEM-1_
SH-73	*S.* Typhimurium	AMP, NAL, TET	*bla*_TEM-1_
SH-75	*S.* Typhimurium	AMP, CIP, NAL, TET	*bla*_TEM-1_
SH-88	*S.* Typhimurium	AMP, CIP, CTX, GEN, NAL, TET	(*drfA1-aadA1)/bla*_TEM-1,_ *bla*_CMY -2_
SH-90	*S.* Typhimurium	AMP, CIP, NAL, TET	*bla*_TEM-1_
SH-93	*S*. Enteritidis	AMP, CIP, NAL, TET	*bla*_TEM-1_
*SH-96*	*S.* Derby	AMP, CIP, NAL, TET	*bla*_TEM-1_

^b^*AMC, amoxicillin/clavulanic acid; AMP, ampicillin; CTX, cefotaxime; CIP, ciprofloxacin; FFC, florfenicol; GEN, gentamicin; NAL, nalidixic acid; SPT, spectinomycin; TET, tetracycline; and SXT, sulfamethoxazole/trimethoprim*.

### Prevalence of Class 1 Integron and β-Lactamase Genes

Among 21 *Salmonella* isolated from CB, class 1 integron was observed in seven isolates (7/21, 33.3%), and gene cassettes included an empty integron (0.15 kb, *n* = 1), *aadA2* (1.2 kb, *n* = 3), *drfA1-aadA1* (1.4 kb, *n* = 1), and *drfA17-aadA5* (1.7 kb, *n* = 2); *bla*_TEM-1_ was the dominant β-lactamase gene (21/21, 100%), followed by *bla*_CTX-M-55_ (7/21, 33.3%) (**Table 1**). Among 19 *Salmonella* isolated from SH, class 1 integron was observed in two isolates (2/19, 10.5%), and gene cassettes included *drfA17-aadA5* (1.7 kb, *n* = 1) and *drfA1-aadA1* (1.4 kb, *n* = 1); *bla*_TEM-1_ was the dominant β-lactamase gene (19/19, 100%), followed by *bla*_CTX-M-55_ (2/19, 10.5%) and *bla*_CMY-2_ (1/19, 5.3%) (**Table 2**). Of note, the proportion of class 1 integron targets detected in *Salmonella* strains from CB samples was higher than that found in SH samples (33.3% vs. 10.5%, *P* < 0.05).

## Discussion

In the present study, 20% of 200 retail chicken carcasses were *Salmonella* positive. The prevalence of *Salmonella* in poultry meat products in other parts of China has been reported by others to be approximately 36.1% (Cui H.X. et al., 2009) and 28.3% (Li et al., 2013). The prevalence in other regions of the world was 15.6% in chicken carcasses in EU (European Food Safety Authority [EFSA], 2010) and 45.8% in retail chicken meat in Korea (Park et al., 2017). These investigations indicated that *Salmonella* contamination is widely distributed in poultry meats. Of note, it is difficult to compare the prevalence of *Salmonella* among different studies, because the difference may be associated with geographical differences, sampling seasons, sample types, methodology of isolation and culture, and environments of slaughterhouses and marketing areas (Yan et al., 2010; Yang et al., 2010).

*Salmonella* Enteritidis was the most commonly isolated serotype in this study, and has been widely isolated in chickens, eggs, and chicken meats in China (Yang et al., 2010; Lu et al., 2011; Long et al., 2016). In addition, *S.* Enteritidis is the leading cause of *Salmonella* related food-borne outbreaks in humans worldwide (Galanis et al., 2006). Of note, *S.* Typhimurium is the main serotype isolated from humans in China (Deng et al., 2012) and *S.* Derby is the most common serotype isolated from infants and toddlers in China (Cui S. et al., 2009), which suggested that an association may exist between *Salmonella*-contaminated food and salmonellosis in these age groups.

Similar antibiotic resistance patterns were observed in *Salmonella* isolated from CB and SH. Of 21 *Salmonella* isolated from CB, high antibiotic resistance rates were found for ampicillin (20/21, 95.2%), nalidixic acid (18/21, 85.7%), cefotaxime (17/21, 81%), and tetracycline (13/21, 61.9%); and 18 out of 21 *Salmonella* were MDR isolates (85.7%). Among 19 *Salmonella* isolated from SH, high antibiotic resistance rates were found for nalidixic acid (19/19, 100%), tetracycline (19/19, 100%), ampicillin (18/19, 94.7%), and ciprofloxacin (13/19, 68.4%); and 17 of 19 *Salmonella* were MDR strains (89.5%). No significant difference (*P* > 0.05) in the prevalence of MDR *Salmonella* between CB and SH. Of note, co-resistance to ciprofloxacin and cefotaxime in these *Salmonella* strains would limit therapeutic options in clinical practice (Whichard et al., 2007).

All class 1 integron-positive isolates in this study exhibited resistance to at least two classes of antibiotics, which supports the hypothesis that there is a strong association between the presence of class I integron and the emerging of MDR in *Salmonella* (Wannaprasat et al., 2011; Firoozeh et al., 2012).

All *Salmonella* isolates in this study carried *bla*_TEM-1_ genes, 38 of which showed were resistant to ampicillin. Noticeably, one *bla*_CMY-2_- producing *Salmonella* isolate was detected, which has been observed in chicken meat in 2010-2011 in Sichuan province of China (Li et al., 2013). Because *bla*_CMY -2_ can encode antibiotic resistance to third-generation cephalosporins, which is frequently used to treat cases of salmonellosis (Gonzalez-Sanz et al., 2009), the dissemination of *bla*_CMY-2_- positive *Salmonella* via poultry meat products has pivotal public health implications. Therefore, the poultry industry should follow prudent management by establishing more effective disinfection guidelines to reduce the population of antibiotic-resistant pathogens. Moreover, a moderate use of antibiotics may help prevent the occurrence of antibiotic resistance in pathogens (Chen and Jiang, 2014).

## Conclusion

To our best knowledge, this is the first study in China comparing the prevalence and characteristics of *Salmonella* isolated from chicken meat samples of CB and SH. Regardless of chicken meat type, 25% (10/40) of the *Salmonella* isolates in this study carried ESBL-producing genes; 22.5% (9/40) of the *Salmonella* isolates contained class 1 integrons. Therefore, the reasonable use of antibiotics in animal husbandry should be taken, and continued long-term surveillance of *Salmonella* in animal-derived foods is warranted.

## Author Contributions

ZM designed the study; SL and YZ collected samples and conducted the experiments; ZM, SL, and YZ analyzed data and wrote the manuscript.

## Conflict of Interest Statement

The authors declare that the research was conducted in the absence of any commercial or financial relationships that could be construed as a potential conflict of interest.

## Footnotes

1 http://www.ncbi.nlm.nih.gov/BLAST
